# Oocyte size, egg index, and body lipid content in relation to body size in the solitary bee *Megachile rotundata*

**DOI:** 10.7717/peerj.314

**Published:** 2014-03-25

**Authors:** Kevin M. O’Neill, Casey M. Delphia, Ruth P. O’Neill

**Affiliations:** 1 Department of Land Resources and Environmental Sciences, Montana State University, Bozeman, MT, USA; 2 Department of Plant Sciences and Plant Pathology, Montana State University, Bozeman, MT, USA

**Keywords:** Hymenoptera, Megachilidae, Egg size, Nesting biology

## Abstract

Females of solitary, nest-provisioning bees have relatively low fecundity, but produce large eggs as part of their overall strategy of investing substantially in each offspring. In intraspecific comparisons of several species of solitary, nest-provisioning bees and wasps, the size of the mature eggs produced increases with female body size. We further examined oocyte size–body size correlations in the solitary bee *Megachile rotundata* (F.), an important crop pollinator. We hypothesized that larger females carry larger basal oocytes (i.e., those next in line to be oviposited) but that body size–oocyte size correlations would be absent soon after emergence, before their first eggs fully matured. Because egg production is likely affected by the quantity of stored lipids carried over from the bees’ immature stages, we also tested the hypothesis that female body size is correlated with the body lipid content at adult emergence, the time during which oocyte growth accelerates. We found significant correlations of body size with oocyte size variables chosen to reflect: (1) the magnitude of the investment in the next egg to be laid (i.e., the length and volume of the basal oocyte) and (2) the longer term potential to produce mature oocytes (i.e., the summed lengths and volumes of the three largest oocytes in each female). Positive correlations existed throughout the nesting season, even during the first week following adult emergence. The ability to produce and carry larger oocytes may be linked to larger females starting the nesting season with greater lipid stores (which we document here) or to greater space within the abdomen of larger females. Compared to other species of solitary bees, *M. rotundata* appears to have (1) smaller oocytes than solitary nest-provisioning bees in general, (2) comparable oocyte sizes relative to congeners, and (3) larger oocytes than related brood parasitic megachilids.

## Introduction

In solitary nest-provisioning bees, females provide each offspring with substantial parental investment in the form of a secure cell within a nest, food required to complete development to the adult stage, and a large yolk-filled egg. Body size is one component of female phenotype that has been linked to variation in female reproductive success within species of solitary bees. Size has been positively correlated with fitness through its effects on provisioning rate, provision mass, fecundity, and offspring size and sex ratio (Kim, 1997; Roulston & Cane, 2000; Bosch & Vicens, 2006; Rehan & Richards, 2010; Seidelmann, Ulbrich & Mielenz, 2010). Thus, factors that affect body size during development can affect reproductive success later in life. Heritability for body size is apparently zero or low for solitary bees (Tepedino, Thompson & Torchio, 1984; Frohlich & Tepedino, 1986; Owen & McCorquodale, 1994). Instead, body sizes attained by adult females are strongly influenced by environmental conditions during larval development, including (1) the amount of pollen and nectar they received (Roulston & Cane, 2000; Radmacher & Strohm, 2010), (2) temperature (Tepedino & Parker, 1986; Radmacher & Strohm, 2010), and (3) tunnel diameter of natal nests of cavity-nesting bees (O’Neill et al., 2010).

Larsson (1990) has further hypothesized that the quantity of nutrients within eggs of solitary nest-provisioning bees and wasps may affect early larval mortality and the body size attained by adult offspring. This is supported with data from sexually-dimorphic solitary wasps in which eggs that produce females are larger on average than eggs that produce males, the smaller sex (Jayasingh, 1980; Budrienė, Budrys & Nevronytė, 2013; but see Strohm, 2000). Egg sizes of solitary bees have been determined primarily with the goal of testing interspecific comparative hypotheses (Iwata, 1955; Iwata & Sakagami, 1966; Alexander & Rozen, 1987; Rozen, 2003). The result has been an emphasis on obtaining data from a large number of species, rather than on securing larger sample sizes needed to make intraspecific comparisons. Nevertheless, in a few species of solitary bees it is known that larger mothers carry larger oocytes or lay larger eggs (Larsson, 1990; Tengo & Baur, 1993; Kim, 1997).

To further test the hypothesis that larger females carry larger oocytes, we examined the relationship of female body size to oocyte size in the alfalfa leafcutting bee, *Megachile rotundata* (F.). *Megachile rotundata* nests in existing cavities, including those in artificial nest shelters placed in alfalfa fields where it is an important commercially-managed pollinator (e.g., Pitts-Singer & Cane, 2011). Because such fields contain large bee populations, we were easily able to collect large enough samples of nesting *M. rotundata* females to test several hypotheses. First, we hypothesized that larger females carry larger basal oocytes, those that are next in line to be oviposited. Second, the female size–oocyte size correlation should be stronger when we consider only the largest oocytes produced by each female size class. This was done to reduce variation introduced by including in the analysis females that have just laid their most mature eggs. Third, we predicted that the female size - oocyte size correlation would be absent soon after females emerged and appear only later when their ovaries were fully developed and space was more limited in their abdomens. We discuss the results relative to previous interspecific comparative studies, and argue for obtaining larger sample sizes for all species, when possible. Lastly, given the fact that stored lipids from fat bodies are important for egg production in insects (Arrese & Soulages, 2010), we also tested the hypothesis that female body size is correlated with the lipid content of their bodies at adult emergence, the time in their lives when oocyte growth accelerates (Richards, 1994).

## Materials and Methods

We collected bees for examination of ovaries at a commercial seed alfalfa farm ∼3 km west of Laurel, MT (45°39′10.99″N, 108°49′39.36″W). The cooperating grower placed trays of bee cells in nest shelters on 20 June 2012 after having incubated them at ∼30 °C since early June. At release, the trays contained of a mix of (1) cells from which adults had already emerged (mostly males in this protandrous species) and (2) cells containing bees in the final stages of pupal-adult development. By the time of our first sampling date on 22 June, many females were taking nesting materials into nest holes to construct leaf-lined nest cells (Pitts-Singer & Cane, 2011). Thus, the majority of females collected had either started nesting or were searching for suitable nest sites. Other samples were taken on 29 June, 6 July, 20 July, and 3 August. We used a sweep net to collect females active at the faces of the nest boards. All females collected were immediately placed in a cooler with ice; after they were returned to the lab later the same day, they were then placed in Kahle’s solution to fix the tissues.

Females collected on each date were removed from the Kahle’s solution and dissected under ethanol using a stereomicroscope equipped with an ocular micrometer; they were in the ethanol for fewer than five minutes, during which time we observed no shrinkage of materials in the oocytes away from the surrounding chorion (membrane). Females were chosen for dissection to span the greatest possible range of head widths available in the samples for each date. For each female, we recorded its head width (HW; to the nearest 0.5 mm). Head width is a significant predictor of dry body mass of females (linear regression, *F* = 990.0, d.f. = 1, 312, *r*^2^ = 0.76, *N* = 314, *P* <0.001; unpublished data from study described in O’Neill et al., 2011). Upon dissection of the females, we measured the length (*L*) and diameter (*D*, at the midpoint of its long axis) of each of the three longest oocytes. If the oocyte was somewhat flattened by being pressed against the inner wall of the exoskeleton, we averaged the value between the greatest and smallest width to get the midpoint value. We also recorded whether the largest oocyte had reached its mature or near-mature sausage shape, a cylinder with hemispherical ends.

For sausage-shaped basal oocytes, we estimated their volume (*V*) in mm^3^ as *V* = (*πr*^2^)(*L*−2*r*) + (4/3)(*πr*^3^), where *r* = *D*/2. Less-developed oocytes approximated a prolate spheroid in shape, so we estimated their volume as *V* = (4/3*πr*^2^)(*L*/2). We used linear regressions to relate HW to (1) the length of the longest (the basal) terminal oocyte (*L*_basal_), (2) the lengths of the second (*L*_2_) and third longest (*L*_3_) oocytes, (3) the summed length of the three measured oocytes (*L*_total_), (4) the volume of the basal oocyte (*V*_basal_), and (5) the summed volumes of the three measured oocytes (*V*_total_); all oocyte variables were transformed as the square root of the value plus 0.5 to satisfy the assumptions of normality and homogeneity of variance (Zar, 1999). For certain regressions, we excluded data from the first sample of the season (22 June) as these females were collected when apparently none had mature or near-mature eggs, based on their shapes and small size.

In previous studies of nest-provisioning and brood-parasitic aculeates (e.g., Iwata, 1955; Iwata & Sakagami, 1966; Alexander & Rozen, 1987; Ohl & Linde, 2003; Rozen, 2003), the “egg index” has been used for interspecific comparisons of relative oocyte size. The egg index, which is calculated as oocyte length/thorax width measured at the outer edges of the tegulae, standardizes oocyte size among insects with vastly different body sizes (Iwata & Sakagami, 1966). Although use of the thorax width rather than head width is a superior measure for interspecific comparisons, we used head width for our intraspecific analyses because it is less ambiguously measured due to the fact that the tegulae become set in different positions in preserved specimens. However, in order to compare our results to studies that used the egg index, we also estimated thorax width in the manner described by Iwata & Sakagami (1966). Using a separate set of bees not used for the oocyte analyses, we regressed thorax width (TW) on HW of pinned *M. rotundata* females representing a broad range of head widths. The resulting regression (TW = 1.081 HW–0.509, *r*^2^ = 0.98, *N* = 30) was used to estimate the TW and the egg index (*L*_basal_/TW) for all of the dissected specimens.

As part of another study to determine the effect of post-overwintering rearing temperatures on *M. rotundata*, we estimated the total body lipid content for 314 newly-emerged females. The source of the bees, the rearing methods, and the technique for estimating lipid content are detailed in (O’Neill et al., 2011). Briefly, to estimate total lipid mass, we calculated the difference between the dry weights of bees before and after being soaked for 10 days in petroleum ether (i.e., a non-polar solvent used for lipid extraction). Here, we used data collected on newly-emerged females from that study (but not previously reported) to relate head width to (1) total lipid mass and (2) the proportion of dry masses of females consisting of lipids (*P_L_*) with the *P_L_* values being first transformed as the arcsine of the square root of *P_L_* to satisfy the assumptions of normality and homogeneity of variance (Zar, 1999).

## Results

As also reported by Richards (1994), all females had three ovarioles in each of two ovaries. Ovaries were located in the posterior half of the metasoma, where they shared the limited space with other organs, including the midgut, hindgut, Malpighian tubules, and poison gland/sting apparatus. The crop occupied most of the anterior half of the metasoma. The interior cavity of the metasoma, particularly when the crop was inflated, was sometimes so crowded that larger oocytes were pressed against the inner surface of the exoskeleton and had indentations conforming to junctions of the segments. In all but one of 145 females, the longest and third-longest oocytes were in one ovary, with the second longest in the other ovary.

**Figure 1:**
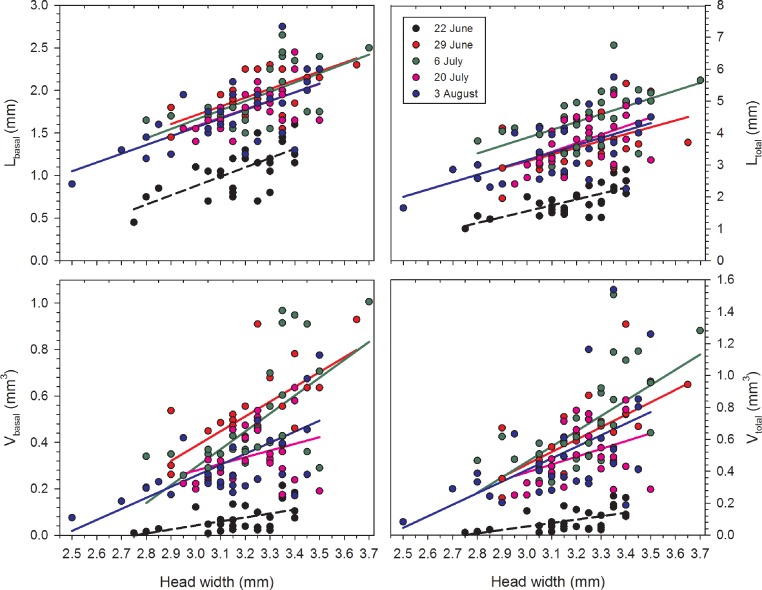
Regressions of oocyte variables on head width of female *Megachile rotundata* (Fabricius, 1787) (Hymenoptera: Megachilidae) for each of the five sampling dates. Regression statistics for analyses of square-root transformed oocyte variables are given in Table 1. Abbreviations are as follows: *L*_basal_, length of the basal oocyte; *V*_basal_, volume of the basal oocyte; *L*_total_, summed length of the basal oocyte plus the second and third longest oocytes; and *V*_total_, summed volumes of the basal oocyte plus the second and third longest oocytes.

Correlations between HW and each of the four variables were positive and significant for each date with the single exception of *V*_basal_ on 20 July (Table 1; Fig. 1). Females on 22 June had relatively undeveloped terminal oocytes, and therefore all oocyte variables were consistently smaller when compared to samples collected later in the summer. The correlations of HW to oocyte size were also significant for the lengths and volumes of second- and third-largest oocytes, although the *r*^2^ values were smaller.

**Table 1:** Linear regressions of square-root transformed oocyte size variables on head width of female *M. rotundata*.

Variable	Date	*N*	*F*	d.f.	*r* ^2^	*P*
*L*_basal_	22 June	30	24.5	1, 28	0.47	<0.001
	29 June	26	16.8	1, 24	0.41	<0.001
	6 July	32	18.9	1, 30	0.39	<0.001
	20 July	27	11.9	1, 25	0.32	<0.01
	3 August	30	21.7	1, 28	0.44	<0.001
	All after 22 June	115	83.1	1, 113	0.42	<0.001
*L*_2_	All after 22 June	115	9.1	1, 113	0.07	<0.01
*L*_3_	All after 22 June	115	23.4	1, 113	0.17	<0.001
*L*_total_	22 June	30	23.2	1, 28	0.45	<0.001
	29 June	26	7.5	1, 24	0.24	<0.05
	6 July	32	12.4	1, 30	0.29	<0.001
	20 July	27	10.0	1, 25	0.29	<0.01
	3 August	30	17.2	1, 28	0.38	<0.001
	All after 22 June	115	48.9	1, 113	0.30	<0.001
*V*_basal_	22 June	30	10.6	1, 28	0.27	<0.01
	29 June	26	17.2	1, 24	0.42	<0.001
	6 July	32	18.6	1, 30	0.38	<0.001
	20 July	27	2.9	1, 25	0.10	= 0.10
	3 August	30	13.9	1, 28	0.33	<0.001
	All after 22 June	115	57.7	1, 113	0.34	<0.001
*V*_2_	All after 22 June	115	13.7	1, 113	0.11	<0.001
*V*_3_	All after 22 June	115	18.3	1, 113	0.14	<0.001
*V*_total_	22 June	30	12.3	1, 28	0.31	<0.001
	29 June	26	17.0	1, 24	0.41	<0.001
	6 July	32	18.1	1, 30	0.38	<0.001
	20 July	27	4.5	1, 25	0.15	<0.05
	3 August	30	12.8	1, 28	0.31	<0.001
	All after 22 June	115	57.4	1, 113	0.34	<0.001
*L*_basal max_ ^*^	All after 22 June	19	74.7	1, 17	0.82	<0.001
*L*_total max_ ^*^	All after 22 June	19	23.9	1, 17	0.59	<0.001
*V*_basal max_ ^*^	All after 22 June	19	69.1	1, 17	0.80	<0.001
*V*_total max_ ^*^	All after 22 June	19	55.1	1, 17	0.76	<0.001

**Notes.**

* Maximum value for each head width class.

**Figure 2:**
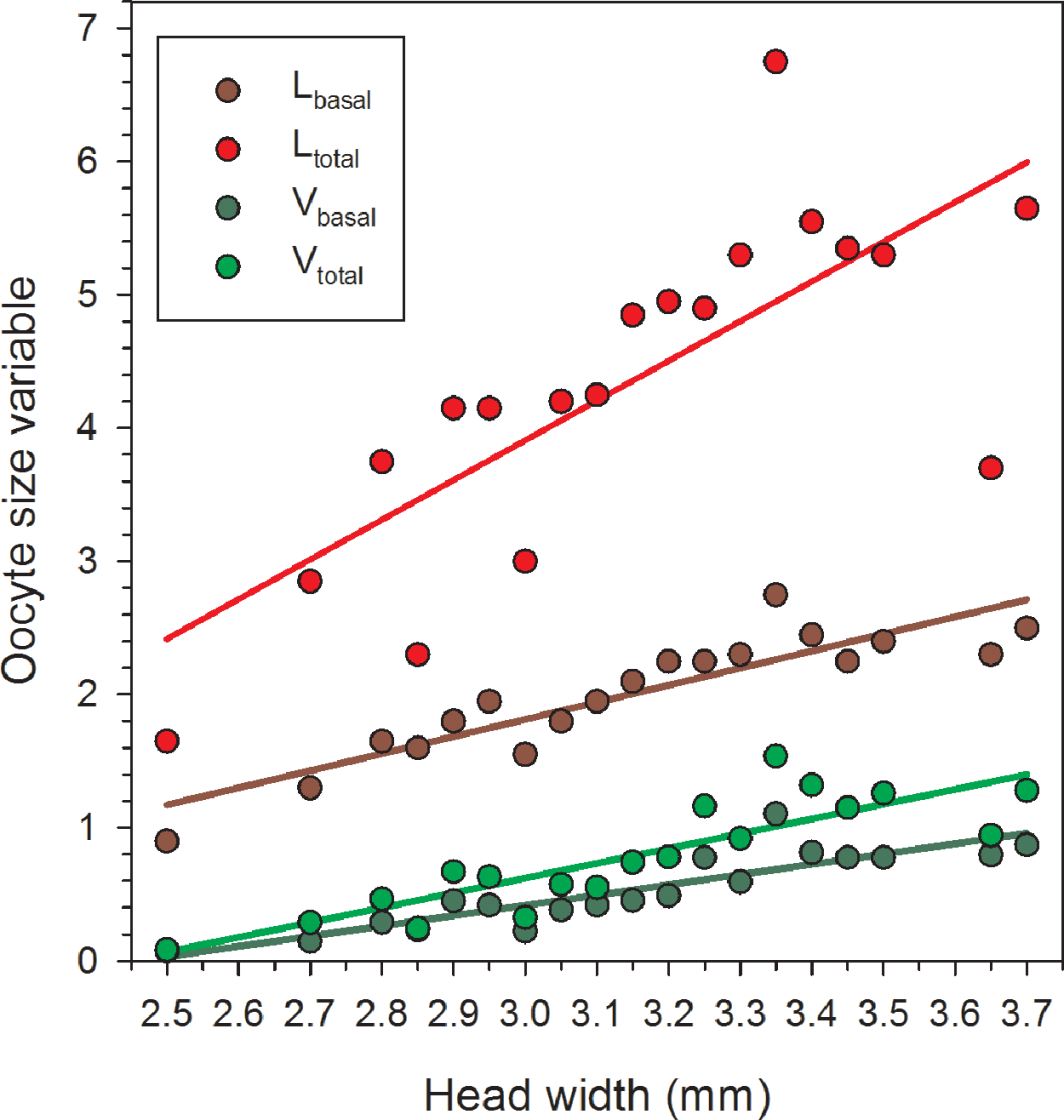
Regressions of oocyte variables on head width of female *Megachile rotundata* (Fabricius 1787) (Hymenoptera: Megachilidae) for each of the five sampling dates, using only the largest values in each head width class (data for 29 June to 3 August only). Regression statistics for analyses of square-root transformed oocyte variables are given in Table 1. Abbreviations are as follows: *L*_basal_, length of the basal oocyte; *V*_basal_, volume of the basal oocyte; *L*_total_, summed length of the basal oocyte plus the second and third longest oocytes; and *V*_total_, summed volumes of the basal oocyte plus the second and third longest oocytes.

Females in the 22 June sample were newly emerged and so likely had had insufficient time to produce mature or near-mature oocytes (Richards, 1994), whereas many females in samples from later in the season had recently laid their largest eggs. As a result, in the overall data set, correlations between HW and the oocyte size variables do not provide an accurate picture of the relationships between body size and the maximum values for *L*_basal_, *L*_total_, *V*_basal_, and *V*_total_. Therefore, we separately regressed the maximum values in each of the nineteen 0.5 mm HW-classes for females collected after 22 June. In these reduced data sets, variation in HW explained the majority of the variation for each of the four oocyte variables (Table 1, Fig. 2).

**Figure 3:**
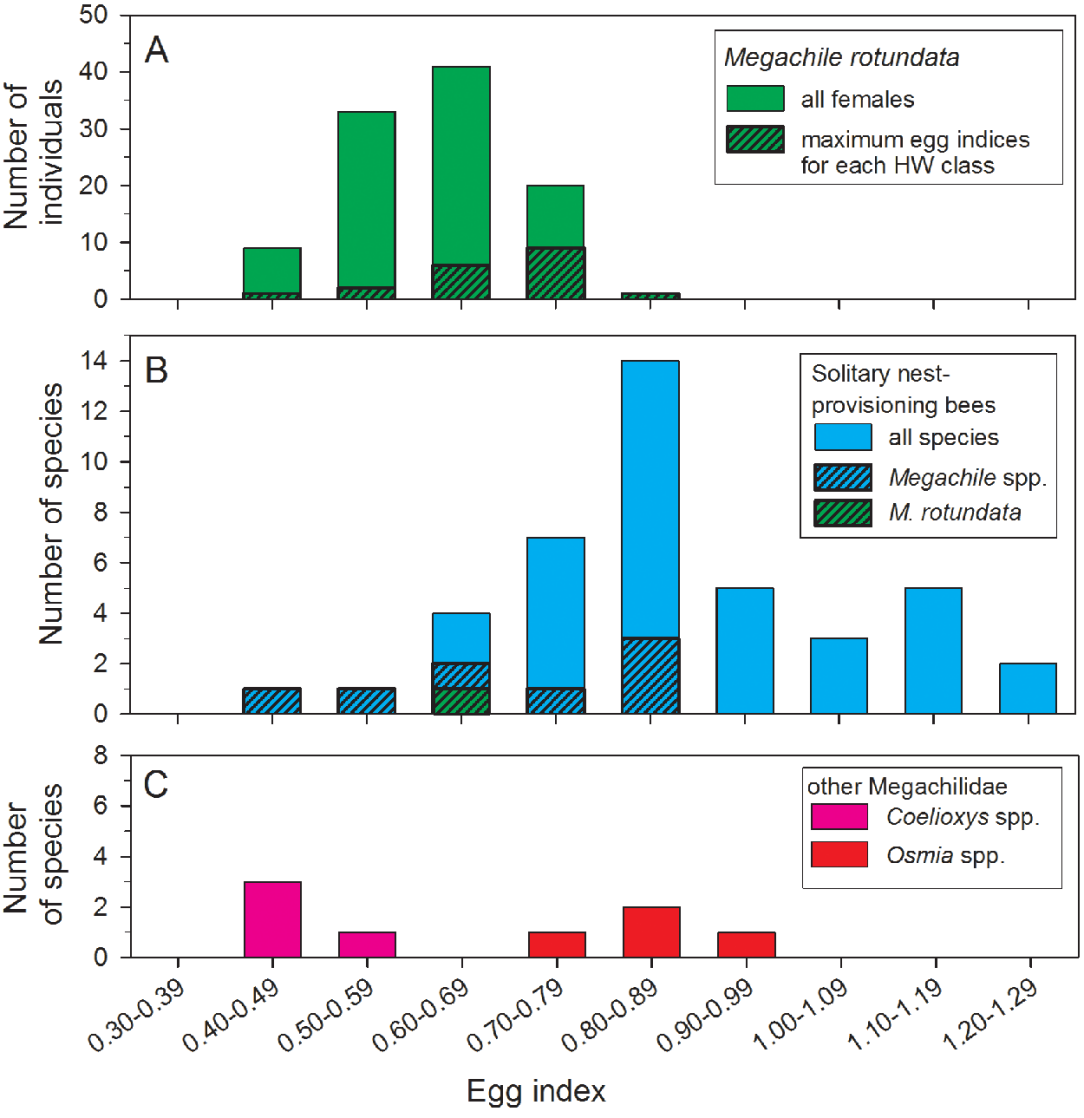
Comparisons of egg index values for female *Megachile rotundata* (Fabricius, 1787) (Hymenoptera: Megachilidae) in this study (A), 41 solitary nest-provisioning bees examined by Iwata & Sakagami (1966), plus the mean *M. rotundata* value (B). Data for *Coelioxys* spp. and *Osmia* spp. are from Iwata & Sakagami (1966) and Rozen (2003). In the top and middle histograms, the black and hatched portions of the bars are subsets of the total height of the bars.

The mean egg index for all females with sausage-shaped *L*_basal_ oocytes collected after 22 June was 0.62 ± 0.01 (*N* = 106) (Fig. 3A). The mean egg index was slightly larger, at 0.68 ± 0.02 (range: 0.41–0.85; *N* = 19), when considering only the maximum *L*_basal_ values for each HW class. Egg indices also varied with body size, there being a significant positive correlation between TW and the square root-transformed egg index values (*r*^2^ = 0.14, *F* = 17.24, d.f. = 1, 104, *P* < 0.001). In other words, larger females have disproportionately larger oocytes on average.

Lab-reared females emerged with lipid contents ranging from 1.7 to 28.3% of their dry mass. In both 2007 and 2008 (and in the combined data set for both years), the total lipid mass and the proportion of dry masses consisting of lipids in newly-emerged females was positively correlated with head width (Table 2). At emergence, smaller females (HW >1 SD below the mean) had about half the lipid mass on average than larger females (HW >1 SD above the mean). The largest females in the samples also had proportionally more lipids than the smallest females, by 20% in 2007, 28% in 2008, and 42% in the combined samples.

**Table 2:** Linear regressions of head width on total lipid mass and proportion body lipids at adult bee emergence (all values based on dry masses). Mann–Whitney tests used to compare values for females with head widths 1 SD ≤ mean versus 1 SD ≥ mean. Year refers to the year that bees emerged.

	Regression of head width on:			Females with head width 1 SD ≤ mean		Females with head width 1 SD ≥ mean		Mann–Whitney test	
Year	*F* (d.f.)	*r* ^2^	*P*	mean ± SE	*N*	mean ± SE	*N*	*U*	*P*
**Total body lipid mass (mg) at emergence**									
2007	108.1 (1, 136)	0.44	<0.001	17.3 ± 1.4	25	32.6 ± 1.6	16	24.0	<0.001
2008	68.4 (1, 174)	0.28	<0.001	13.7 ± 1.0	36	26.9 ± 1.3	30	57.5	<0.001
Both years	242.5 (1, 312)	0.44	<0.001	14.2 ± 1.0	46	31.5 ± 0.9	50	92.5	<0.001
**Proportion dry mass body lipids at emergence**									
2007	15.0 (1, 136)	0.10	<0.001	0.157 ± 0.011	25	0.188 ± 0.008	16	115.0	=0.02
2008	11.5 (1, 174)	0.06	<0.001	0.125 ± 0.008	36	0.164 ± 0.006	30	279.0	<0.001
Both years	57.3 (1, 312)	0.16	<0.001	0.131 ± 0.008	46	0.186 ± 0.004	50	414.0	<0.001

## Discussion

Our results indicate that oocyte size co-varies with female body size in *M. rotundata*. Female size and oocyte size were positively correlated on all collection dates (with the single exception of the HW-*V*_basal_ analysis for 20 July), and in combined data sets for all females collected after the first week of the nesting season. After excluding data for 22 June (thus eliminating most recently-emerged females) and combining those from the other dates, variation in HW explained 42% of the variation in the length and 34% of variation in the volume of the basal oocyte. Because the basal oocyte is the next in line to be deposited on a pollen/nectar mass in a cell, this suggests that larger females provide a greater initial mass of nutrients to their offspring (O’Neill, 1985; Larsson, 1990).

Besides the potential benefit of producing a larger egg, there may also be a value to producing mature eggs at a higher rate. In insects in general, body size has been consistently linked to intraspecific variation in fecundity (Honĕk, 1993). More specifically for nest-building Hymenoptera, Rosenheim, Nonacs & Mangel (1996) predicted that, when provisioning resources were abundant, it is egg maturation rate that limits reproductive success. This limitation has been observed or inferred from indirect evidence in several studies of solitary nesting-provisioning bees including *M. rotundata* (Gerber & Klostermeyer, 1972; Bohart & Youssef, 1976; Danforth, 1989; Minckley et al., 1994; Kim, 1999). When floral resources are available and weather conditions are suitable, female *M. rotundata* can complete one, and sometimes two, cells per day (Klostermeyer & Gerber, 1969; Gerber & Klostermeyer, 1972), but only if they maintain a steady rate of oocyte maturation. Depending on their age, female *M. rotundata* carry an average of 17–38 oocytes of all sizes (Richards, 1994), though many are small and undeveloped. In our study, female body size explained 30% of the variation in the summed length and 34% of the summed volume of the three largest oocytes, partly because the second and third largest oocytes also increased with female size. Thus, because their secondary and tertiary oocytes tend to be larger, larger females may be able to more quickly produce another egg of minimum viable size and shorten the time to the next oviposition. They may also be able to do this while continuing to produce larger eggs than smaller females. The latter is substantiated by the fact that the oocyte size—head width correlations remained significant across the six-week sampling period.

Size- and age-related limitations on the rate of egg production in *M. rotundata* may have implications for the population dynamics of this important commercial pollinator. It is not uncommon for completed cells of *M. rotundata* to contain pollen/nectar masses but no eggs or larvae, a condition referred to as “broodless pollen balls” (Pitts-Singer & James, 2008). Broodless cells tend to appear during periods of cool weather, at which time there may be temporary reductions in floral resource abundance. The resulting delay in cell completion may force females to resorb the basal oocyte, seal off the partially-provisioned eggless cell, and construct a new cell; this assumes that the pollen/nectar mass in the older cell must be used within a set period of time before it is no longer suitable for consumption. The proportion of eggs in the process of being resorbed is higher in times of lower resource abundance in *Osmia* spp. (Maeta & Kurihara, 1971), *Megachile flavipes*, and *Megachile cephalotes* (Sihag, 1986). However, in *M. rotundata* some broodless cells could be attributable to limited rates of egg production in older (Richards, 1994) and smaller females. Testing this hypothesis would require associating females of different ages and sizes with particular nests where the frequency of broodless cells could be quantified.

Contrary to expectations, the correlations between head width and oocyte size variables soon after emergence (22 June) were not only significant, but the regressions for *L*_basal_ and *L*_total_ explained a greater proportion of the variance than those for all females after 22 June. Two hypotheses might explain this result. First, space may be so constrained within the females’ abdomens (O’Neill, 1985) that it influences the space available even for immature oocytes. Those space constraints appear to be a result of space dedicated to the crop, which was often full of either pollen or nectar in dissected females. Second, larger females may be better able to mobilize fat body nutrients for oocyte production early in the post-eclosion period, before further materials can be obtained from feeding on pollen. In this study, larger females carried a greater mass of lipids and a greater proportion of their dry masses consisted of lipids. Oocyte development in newly-emerged *M. rotundata* females coincides with a sharp decline in lipid stores (KM O’Neill, CM Delphia, & T Pitts-Singer, 2012, unpublished data). That decline is not likely due to the use of lipids in flight metabolism because bees fuel flights primarily with dietary carbohydrates (Suarez et al., 2005). In insects in general, however, fat body lipids accumulated during larval development are known to be important as sources of nutrients for egg production (Arrese & Soulages, 2010). *Megachile* also require protein in their diets, obtained via pollen consumption, to initiate oocyte development (Sihag, 1986; Richards, 1994), but the relative importance of fat body materials and dietary nutrients is unknown. Other observations on bees are consistent with the hypothesis that dietary nutrients are required for egg production. In the facultatively social bee *Exoneura robusta* Cockerell, for example, females lay smaller eggs at times of year when resources are shifted towards larval feeding and away from egg production (Kayaalp & Schwarz, 2007).

The data reported here give further evidence that the common (though not universal) link between body size and fitness in female solitary bees (Roulston & Cane, 2000; Bosch & Vicens, 2006; Seidelmann, Ulbrich & Mielenz, 2010; Rehan & Richards, 2010) begins early in the life cycles of their offspring. Female body size is also positively correlated with egg or oocyte size (Larsson, 1990; Tengo & Baur, 1993; Kim, 1997), egg number (Sugiura & Maeta, 1989; Tengo & Baur, 1993), and ovariole number (Richards, 1994) in other solitary bees. Similar correlations occur in solitary apoid wasps (O’Neill, 1985; Larsson, 1990; O’Neill & Pearce, 2007), which are taxonomically-related and behaviorally-similar to solitary bees.

Egg size, as a measure of parental investment, can be compared among species only if variation in body size is taken into account. To do this, Iwata & Sakagami (1966) developed the egg index as a measure of oocyte size relative to thorax width. For *M. rotundata*, we found egg indices ranging from 0.41 to 0.85, with a mean of 0.62, for all females with sausage-shaped oocytes collected after 22 June, and a mean of 0.68 for the subset of females with the largest oocytes in each HW class (Fig. 3A). Iwata & Sakagami (1966) report egg index values for 41 species of solitary nest-provisioning bees (Fig. 3B, which also includes our measurement of the egg index of *M. rotundata*); the mean (based on our calculations) for 42 species (including *M. rotundata*) is 0.87 (range: 0.47–1.25). Eighty-eight percent of the species had egg indices >0.68, so *Megachile rotundata* is on the lower end of the range of values.

This percentage is a tentative estimate, however, because the data of Iwata and Sakagami are based on dissection of just 1–2 individuals in each species. The broad range of values that we found for the egg index of *M. rotundata*, as well as for 30 females of the solitary wasp *Stizus renicinctus* (0.65–0.96; O’Neill & Pearce, 2007), indicate that egg index values based on just a few specimens per species could be misleading due to sampling error. Thirty-four percent of the egg index values for female *M. rotundata* with sausage-shaped oocytes after 22 June were at least one standard deviation from the mean. Assuming that similar variation is also present in other species, dissection of a single randomly-selected bee could often give an inaccurate estimate of the egg index. In some studies, part of the variation is removed by using criteria to assure that only fully-mature oocytes are measured; for example, Rozen (2003) measured eggs only when they had “conspicuous chorions”. But another possible source of variation is the correlation between body size and egg index values: larger female *M. rotundata* tended to have larger egg indices.

Despite the potential shortcomings of some published egg index values for comparisons among pairs of species, such data sets can be valuable for testing general hypotheses about the relationship of parental strategies to oocyte size and number (Ito, 1978; O’Neill, 2001; Rozen, 2003), even if using a large number of species does not fully compensate for within-species sampling errors. Thus, it appears that *M. rotundata* invests relatively less in individual eggs than most other solitary nest-provisioning bees (Fig. 3B). Compared to other solitary nest provisioning bees, the egg indices for eight species of *Megachile* (range: 0.50–0.87) are average or below-average in magnitude (Fig. 3B), even compared to four species of *Osmia*, another genus of nest-provisioning Megachilidae (Fig. 3C). In contrast, *Coelioxys* spp. (brood parasites from the same subfamily as *Megachile*) had lower egg indices than most *M. rotundata* (range: 0.40–0.54; Iwata & Sakagami, 1966). Lower egg indices are expected for brood parasitic aculeate bees (Ito, 1978; Alexander & Rozen, 1987; Rozen, 2003) and wasps (O’Neill, 1985; Ohl & Linde, 2003; Evan & O’Neill, 2007). Brood parasitic bees, which lay their eggs on the nest provisions of bees of other species, benefit from carrying a larger number of mature eggs to take advantage of the possibility of encountering multiple hosts over a short period of time. Thus, due to an egg size—egg number tradeoff, they must necessarily carry smaller oocytes.

In summary, in intraspecific comparisons, we found significant correlations between head width, as a measure of overall body size, and four different oocyte size variables chosen to estimate the magnitude of the investment in the next egg to be laid (*L*_basal_ and *V*_basal_) and the longer term potential for producing mature oocytes (*L*_total_ and *V*_total_). The ability to produce and carry larger oocytes may be linked to greater space within the abdomen of larger females and to their beginning the nesting season with greater lipid stores. Although this suggests that there is ongoing selection for larger body size in females, size in this species is non-heritable (Owen & McCorquodale, 1994); rather, variation in body size in *M. rotundata* populations is likely due mainly to environmental effects, such as the amount of food provided by the mother (Klostermeyer, Mech Jr & Rasmussen, 1973) and the diameter of available nest cavities (O’Neill et al., 2010). In interspecific comparisons, *M. rotundata* appears to have (1) smaller oocytes than solitary nest-provisioning bees in general, (2) comparable oocyte sizes relative to congeners, and 3) larger oocytes than related brood parasitic megachilids. Our results highlight the value of obtaining oocyte measurements from a large number of conspecifics in order to test intraspecific hypotheses and gauge how often published egg index values are representative of a species.

## References

[ref-1] Alexander B, Rozen JG (1987). Ovaries, ovarioles, and oocytes in parasitic bees (Hymenoptera: Apoidea). Pan-Pacific Entomologist.

[ref-2] Arrese EL, Soulages JL (2010). Insect fat body: energy, metabolism, and regulation. Annual Review of Entomology.

[ref-3] Bohart GE, Youssef NN (1976). The biology and behavior of *Evylaeus galpinsiae* Cockerell (Hymenoptera: Halictidae). Wasmann Journal of Biology.

[ref-4] Bosch J, Vicens N (2006). Relationship between body size, provisioning rate, longevity and reproductive success in females of the solitary bee *Osmia cornuta*. Behavioral Ecology and Sociobiology.

[ref-5] Budrienė A, Budrys E, Nevronytė Ž (2013). Sexual size dimorphism in the ontogeny of the solitary predatory wasp *Symmorphus allobrogus* (Hymenoptera: Vespidae). Comptes Rendus Biologie.

[ref-6] Danforth BN (1989). Nesting behavior of four species of *Perdita* (Hymenoptera: Andrenidae). Journal of the Kansas Entomological Society.

[ref-7] Evan HE, O’Neill KM (2007). The sand wasps: natural history and behavior.

[ref-8] Frohlich DR, Tepedino VJ (1986). Sex ratio, parental investment, and interparent variability in nesting success in a solitary bee. Evolution.

[ref-9] Gerber HS, Klostermeyer EC (1972). Factors affecting the sex ratio and nesting behavior of the alfalfa leafcutting bee.

[ref-10] Honĕk A (1993). Intraspecific variation in body size and fecundity in insects: a general relationship. Oikos.

[ref-11] Ito Y (1978). Comparative Ecology.

[ref-12] Iwata K (1955). The comparative anatomy of the ovary in Hymenoptera. Part I. Aculeata. Mushi.

[ref-13] Iwata K, Sakagami SF (1966). Gigantism and dwarfism in bee eggs in relation to modes of life, with notes on the number of ovarioles. Japanese Journal of Ecology.

[ref-14] Jayasingh DB (1980). A new hypothesis on cell provisioning in solitary wasps. Biological Journal of the Linnean Society.

[ref-15] Kayaalp P, Schwarz MP (2007). Egg size and number is influenced by both environmental and social factors in a facultatively social bee. Australian Journal of Zoology.

[ref-16] Kim J-Y (1997). Female size and fitness in the leaf-cutter bee *Megachile apicalis*. Ecological Entomology.

[ref-17] Kim J-Y (1999). Influence of resource level on maternal investment in a leaf-cutter bee (Hymenoptera: Megachilidae). Behavioral Ecology.

[ref-18] Klostermeyer EC, Gerber HS (1969). Nesting behavior of *Megachile rotundata* (Hymenoptera: Megachilidae) monitored with an event recorder. Annals of the Entomological Society of America.

[ref-19] Klostermeyer EC, Mech SJ, Rasmussen WB (1973). Sex and weight of *Megachile rotundata* (Hymenoptera: Megachilidae) progeny associated with provision weights. Journal of the Kansas Entomological Society.

[ref-20] Larsson FK (1990). Female body size relationships with fecundity and egg size in two solitary species of fossorial Hymenoptera (Colletidae and Sphecidae). Entomologia Generalis.

[ref-21] Maeta Y, Kurihara M (1971). Anatomical and histological studies on the oogenesis and oosorption of terminal oocytes within the genus Osmia. Kontyu.

[ref-22] Minckley RL, Wcislo D, Yanega D, Buchmann SL (1994). Behavior and phenology of a specialist bee (*Dieunomia*) and sunflower (*Helianthus*) pollen availability. Ecology.

[ref-23] Ohl M, Linde D (2003). Ovaries, ovarioles, and oocytes in apoid wasps, with special references to cleptoparasitic species (Hymenoptera: Apoidea: Sphecidae). Journal of the Kansas Entomological Society.

[ref-24] O’Neill KM (1985). Egg size, prey size, and sexual dimorphism in digger wasps. Canadian Journal of Zoology.

[ref-25] O’Neill KM (2001). Solitary wasps: behavior and natural history.

[ref-26] O’Neill KM, O’Neill RP, Kemp WP, Delphia CM (2011). Effect of temperature on post-wintering development and total lipid content of alfalfa leafcutting bees. Environmental Entomology.

[ref-27] O’Neill KM, Pearce AM (2007). Ovary structure and oocyte size in relation to female size and age in the brood parasitic wasp *Stizoides renicinctus* (Say) (Hymenoptera: Crabronidae). Proceedings of the Entomological Society of Washington.

[ref-28] O’Neill KM, Pearce AM, O’Neill RP, Miller RS (2010). Offspring size and sex ratio variation in a feral population of alfalfa leafcutting bees (Hymenoptera: Megachilidae). Annals of the Entomological Society of America.

[ref-29] Owen RE, McCorquodale DB (1994). Quantitative variation and heritability of post-diapause development time and body size in the alfalfa leafcutting bee (Hymenoptera: Megachilidae). Annals of the Entomological Society of America.

[ref-30] Pitts-Singer TL, Cane JH (2011). The alfalfa leafcutting bee, *Megachile rotundata*: the world’s most intensively managed solitary bee. Annual Review of Entomology.

[ref-31] Pitts-Singer TL, James RR (2008). Do weather conditions correlate with findings in failed, provision-filled nest cells of *Megachile rotundata* (Hymenoptera: Megachilidae) in western North America?. Journal of Economic Entomology.

[ref-32] Radmacher S, Strohm E (2010). Factors affecting offspring body size in the solitary bee *Osmia bicornis* (Hymenoptera, Megachilidae). Apidologie.

[ref-33] Rehan S, Richards MH (2010). The influence of maternal quality on brood sex allocation in the small carpenter bee, *Ceratina calcarata*. Ethology.

[ref-34] Richards KW (1994). Ovarian development in the alfalfa leafcutter bee, *Megachile rotundata*. Journal of Apicultural Research.

[ref-35] Rosenheim JA, Nonacs P, Mangel M (1996). Sex ratios and multifaceted parental investment. American Naturalist.

[ref-36] Roulston TH, Cane JH (2000). The effect of diet breadth and nesting ecology on body size variation in bees (Apiformes). Journal of the Kansas Entomological Society.

[ref-37] Rozen JG (2003). Eggs, ovarioles numbers, and modes of parasitism of cleptoparasitic bees, with emphasis on neotropical species. American Museum Novitates.

[ref-38] Seidelmann K, Ulbrich K, Mielenz N (2010). Conditional sex allocation in the red mason bee, *Osmia rufa*. Behavioral Ecology and Sociobiology.

[ref-39] Sihag RC (1986). Reproduction in alfalfa pollinating sub-tropical megachilid bees. 4. Vitellogenesis and oosorption, and factors inducing these processes. Zoologischer Anzeiger.

[ref-40] Strohm E (2000). Factors affecting the body size and fat content in a digger wasp. Oecologia.

[ref-41] Suarez RK, Darveau C-A, Welch KC, O’Brien DM, Roubik DW, Hochachka PW (2005). Energy metabolism in orchid bee flight muscles: carbohydrate fuels all. Journal of Experimental Biology.

[ref-42] Sugiura N, Maeta Y (1989). Parental investment and offspring sex ratio in a solitary Mason bee, *Osmia cornifrons* (Radoszkowski) (Hymenoptera: Megachilidae). Japanese Journal of Entomology.

[ref-43] Tengo J, Baur B (1993). Number and size of oocytes in relation to body size and time of day in the kleptoparasitic bee *Nomada lathburiana* (Hymenoptera: Anthophoridae). Entomologia Generalis.

[ref-44] Tepedino VJ, Parker FD (1986). Effect of rearing temperature on mortality, second-generation emergence, and size of adult is *Megachile rotundata* (Hymenoptera: Megachilidae). Journal of Economic Entomology.

[ref-45] Tepedino VJ, Thompson R, Torchio PF (1984). Heritability for size in the megachilid bee *Osmia lignaria propinqua*. Apidologie.

[ref-46] Zar JH (1999). Biostatistical Analysis.

